# High Incidence of Cranial Synostosis and Chiari I Malformation in Children With X‐Linked Hypophosphatemic Rickets (XLHR)

**DOI:** 10.1002/jbmr.3614

**Published:** 2018-11-20

**Authors:** Anya Rothenbuhler, Nathalie Fadel, Yahya Debza, Justine Bacchetta, Mamadou Tidiane Diallo, Catherine Adamsbaum, Agnès Linglart, Federico Di Rocco

**Affiliations:** ^1^ APHP, Reference Center for Rare Disorders of the Calcium and Phosphate Metabolism Filière OSCAR Bicêtre Paris Sud Hospital Le Kremlin Bicêtre France; ^2^ APHP, Endocrinology and Diabetes for Children Bicêtre Paris Sud Hospital Le Kremlin Bicêtre France; ^3^ Plateforme d'Expertise Maladies Rares Paris‐Sud Bicêtre Paris Sud Hospital Le Kremlin Bicêtre France; ^4^ APHP, Department of Pediatric Radiology Bicêtre Paris Sud Hospital Le Kremlin Bicêtre France; ^5^ Pediatric Nephrology Hôpital Femme Mère Enfant Hospices Civiles de Lyon and University Claude Bernard Lyon 1 Bron Cedex France; ^6^ EA 2496 Pathologies, Imaging, and Biotherapies of the Tooth Dental School Université Paris Descartes Sorbonne; APHP Odontology Department, Groupement Hospitalier Nord Val de Seine (Bretonneau) Paris France; ^7^ INSERM U1185 and Paris Sud Paris‐Saclay University Bicêtre Paris Sud Hospital Le Kremlin Bicêtre France; ^8^ Pediatric Neurosurgery, Hôpital Femme Mère Enfant Hospices Civiles de Lyon and University Claude Bernard Lyon 1 Bron Cedex France; ^9^ Reference Center for Craniosynostosis INSERM 1033 Lyon France

**Keywords:** X‐LINKED HYPOPHOSPHATEMIC RICKETS (XLHR), CHIARI TYPE I MALFORMATION, SCAPHOCEPHALY, CRANIOSYNOSTOSIS

## Abstract

X‐linked hypophosphatemic rickets (XLHR) represents the most common form of genetic hypophosphatemia and causes rickets and osteomalacia in children because of increased FGF23 secretion and renal phosphate wasting. Even though cranial vault and craniovertebral anomalies of potential neurosurgical interest, namely early closure of the cranial sutures and Chiari type I malformation, have been observed in children with XLHR, their actual incidence and characteristics are not established. The aims of this study were to analyze the incidence of cranial and cervico‐occipital junction (COJ) anomalies in children with XLHR and describe its features. This is a retrospective study of CT scans of the head and skull in 44 XLHR children followed at the French Reference Center for Rare Diseases of the Calcium and Phosphate Metabolism. Forty‐four children with XLHR, 15 boys and 29 girls, aged 8.7 ± 3.9 years at time of CT scan, were studied. We found that 59% of XLHR children had a complete or partial fusion of the sagittal suture and 25% of XLHR children showed protrusion of the cerebellar tonsils. A history of dental abscesses was associated with craniosynostosis, and craniosynostosis was associated with abnormal descent of cerebellar tonsils. Only 2 patients showed neurologic symptoms. Four of 44 patients (9%) required neurosurgery. This study highlights that sagittal suture fusion and Chiari type I malformation are frequent complications of XLHR. The incidence of sagittal synostosis in XLHR is actually extremely high and was probably underestimated so far. Chiari type I malformation is also frequent. Because diagnosis of craniovertebral anomalies can be underestimated on a purely clinical basis, radiological studies should be considered in XLHR children if a proper diagnosis is warranted. © 2018 The Authors. *Journal of Bone and Mineral Research* Published by Wiley Periodicals, Inc.

## Introduction

X‐linked hypophosphatemic rickets (XLHR, OMIM 307800) due to inactivating mutations in the PHEX gene (Phosphate Regulating Gene with Homologies to the Endopeptidase on the X chromosome MIM #300550) is the most common form of genetic hypophosphatemic rickets with an incidence of 1 in 20,000.^1^, ^2^

PHEX is expressed in osteocytes and odontoblasts, and inactivating mutations result in increased synthesis and secretion of fibroblast growth factor 23 (FGF23). Part of the physiopathological mechanism that underlies XLHR is impaired proximal renal phosphate reabsorption and reduced 1,25(OH)2D levels both due to the excess action of (FGF23). FGF23 suppresses transcription of the sodium‐phosphate cotransporter genes in the proximal renal tubule cells. FGF23 also downregulates CYP271B (gene that encodes 25‐OHD‐1 alpha hydroxylase) and upregulates CYP24A1 (gene that encodes the 24 hydroxylase) leading to decreased synthesis and increased catabolism of 1,25(OH)2D.^3^, ^4^, ^5^, ^6^, ^7^, ^8^, ^9^

The intrinsic role of PHEX on bone and teeth mineralization remains unclear.

XLHR in children is characterized by progressive leg bowing, which appears when toddlers start to stand and walk, a particular style of walking with a waddling gait, lower‐extremity deformities, poor growth that leads to short stature, morphological changes in the shape of the skull, radiographic changes of rickets, and dental abscesses. Adults with XLHR present with osteomalacia. They may have spontaneous insufficiency fractures, arthritis, enthesopathies (calcifications of tendons and ligaments), and disabling skeletal pain. Children with XLHR present with renal phosphate wasting, ie, hypophosphatemia and impaired renal tubular phosphate reabsorption, elevated serum alkaline phosphatase (ALP), inappropriately low 1,25(OH)2D levels, normal or moderately elevated PTH levels, and normal serum calcium and 25OHD levels.^10^, ^11^, ^12^, ^13^

XLHR children are at risk to develop cranial vault and craniovertebral anomalies of potential neurosurgical interest, namely early closure of the cranial sutures and Chiari type 1 malformation. Though the association of craniosynostosis to rickets has been documented as early as 1964 and several reports of scaphocephaly in patients with rickets have been described,^16^ the literature on craniosynostosis and Chiari type I malformations occurring in patients with XLHR remains scarce, limited only to some case reports and very few case series.^14^, ^15^, ^16^, ^17^, ^18^, ^19^, ^20^, ^21^, ^22^, ^23^, ^24^, ^25^

The aim of our retrospective study was to determine the incidence and characteristics of cranial and craniovertebral anomalies in a large series of children with X‐linked hypophosphatemic rickets.

## Patients and Methods

### Patients

Forty‐four children with XLHR followed at the French National Reference Center for Rare Diseases of the Calcium and Phosphate Metabolism were considered for this retrospective study. The diagnosis of XLHR was based on the typical biochemical features, on disease transmission, and, in most cases, genetic analysis. We collected clinical and biochemical data from patients’ medical records, including age at diagnosis, age at start of treatment and at CT scan evaluation, genetic diagnosis, mode of inheritance, height, and ALP and phosphate level.

The study was conducted according to the French regulations and approved by the French National Commission on Informatics and liberty (CNIL). The need for written consent is waived by French regulations in this type of retrospective study. Parents were orally informed of the study and their consent obtained. They have a right to withdraw from the study at any time by sending a correspondence to http://recherche.aphp.fr/eds/droit-opposition.

### Methods

Brain/skull CTs that had been performed on a 64‐slice CT scanner were studied to assess the patency of the sutures. CT scans were performed only if children were able to lie down with no movement for 20 minutes without anesthesia. CT scans were interpreted independently by two pediatric radiologists and one pediatric neurosurgeon. Radiation doses received by the patients were measured in dose length product (DLP) and ranged between 333 and 550 mGy.cm according to the current guidelines.

Imaging criteria were evaluated as follows:

The patency of the sutures (sagittal, coronal, and lambdoid sutures) was assessed on 3D reconstructions and on thin bone window. In case of fusion of a suture, complete or partial closure was defined as >50% or <50%. The cranial index (CI) was calculated as follows: CI (%) = (Transverse diameter / A‐P diameter) × 100 using the 3D reconstruction of the skull. A cranial index <75% indicates skull deformity. Mesocephaly is defined by a cranial index between 75% and 80%.

The measurement of the frontal cranial vault thickness was performed using bone window and maximum intensity projection on sagittal reconstructions taking both inner and outer table of the cortical bone. The subarachnoid spaces were assessed by measuring the distance between the frontal cortex and the inner table of the skull on frontal reconstructions. The visibility (versus compression) of the sulci was noted.

The ventricles were considered dilated when the diameter of the atria measured ≥10 mm on frontal reconstructions.

The sella turcica was assessed using the anteroposterior diameter and the height on sagittal reconstructions. It was considered enlarged when >10 mm.

The position of the cerebellar tonsils was studied on sagittal reconstructions. A Chiari type 1 malformation was defined by a caudal descent of the cerebellar tonsils through the foramen magnum. A protrusion of the cerebellar tonsils was defined as a descent <5 mm.

The position of the bulbo‐medullary junction, above or under the foramen magnum, was noted.

### Statistics

Clinical, radiological, and biochemical data were compared. Statistical analyses were carried out using GraphPad Prism (v6, GraphPad, La Jolla, CA, USA). All values are presented as means ± SD. The significance threshold was set at *p* < 0.05.

## Results

### Patients

Forty‐four children (15 boys and 29 girls) with XLHR were studied. Thirty‐one children had a familial disease (10 boys and 21 girls) and 13 had de novo XHLR (5 boys and 8 girls). We found a *PHEX* mutation in 36 (82%) XLHR children.

XLHR was diagnosed between 2001 and 2015. Age at diagnosis and treatment initiation was 2.2 ± 2.3 years (*n* = 31, range 0.2 to 10.2 years) in familial XLHR and 3.1 ± 1.4 years (*n* = 13, range 1.5 to 6.3 years) in de novo XLHR.

Age at head and skull CT imaging was 8.7 ± 3.9 years (*n* = 44, range 0.4 to 19 years).

Clinical and biochemical characteristics of the patients at diagnosis and at CT scan evaluation are detailed in Tables 1 and 2.

**Table 1:** Patients’ Characteristics at Diagnosis of XLHR and Treatment Initiation

	At diagnosis	*n*
Boys/girls	15/29	44
Familial/de novo	31/13	44
PHEX mutation (yes/no)		
All	36/8	44
Familial	25/6	31
De novo	11/2	13
ALP (IU/L), mean ± SD (min to max)	560 ± 176 (291 to 1044)	35
Serum phosphate (mmol/L), mean ± SD (min to max)	0.87 ± 0.18 (0.53 to 1.42)	38
Age (years), mean ± SD (min to max)		
All	2.4 ± 2.1 (0.2 to 10.2)	44
Familial	2.2 ± 2.3 (0.2 to 10.2)	31
De novo	3.1 ± 1.4 (1.5 to 6.3)	13
Height (*Z*‐score), mean ± SD (min to max)	–1.13 ± 1.25 (–3.6 to 2)	44

XLHR = X‐linked hypophosphatemic rickets; ALP = alkaline phosphatase.

**Table 2:** Patients’ Characteristics at Time of Head and Skull CT

	At CT scanner	*n*
Age (years), mean ± SD (min to max)	8.7 ± 3.9 (0.4 to 19)	44
Height (*Z*‐score), mean ± SD (min to max)	−0.88 ± 1.2 (–3.8 to 1.5)	44
BMI (*Z*‐score), mean ± SD (min to max)	0.52 ± 1.1 (–0.77 to 5.1)	43
ALP (IU/L), mean ± SD (min to max)	374 ± 127 (168 to 712)	42
History of dental abscesses (yes/no)	20/24	44
Leg bowing (yes/no)	31/13	44

BMI = body mass index; ALP = alkaline phosphatase.

General neurological examination was within normal limits for age except for 2 patients. One adolescent patient with craniosynostosis and a Chiari type I malformation had neurological symptoms (neck pain exacerbated by Valsava maneuvers) after mild head trauma due to a bike accident, and a 8‐year‐old girl with craniosynostosis and a Chiari type I malformation had chronic headaches and attention deficit disorder.

Four of 44 patients required neurosurgery (9%): the 2 above‐mentioned symptomatic patients and 2 additional asymptomatic patients, a 2.75‐year‐old girl at time of CT (which showed a Chiari malformation but no craniosynostosis) whose head and spine MRI showed increasing cervical and thoracic syringomyelia during 2‐year follow‐up and a 5.4‐year‐old boy at time of CT (which showed sagittal craniosynostosis and a Chiari malformation) whose head and spine MRI showed pan medullary cervical and thoracic syringomyelia.

### Craniosynostosis

**Figure 1:**
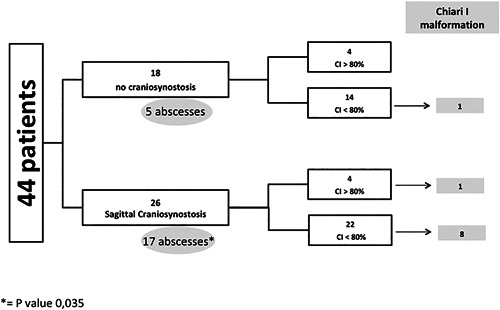
Study population flow chart. CI = cranial index.

Craniosynostosis was found on CT scans in 26 XLHR children (59%). In all cases, it involved the sagittal suture, which was completely fused in 18 cases and partially fused in 8 cases (Fig. 1).

**Figure 2:**
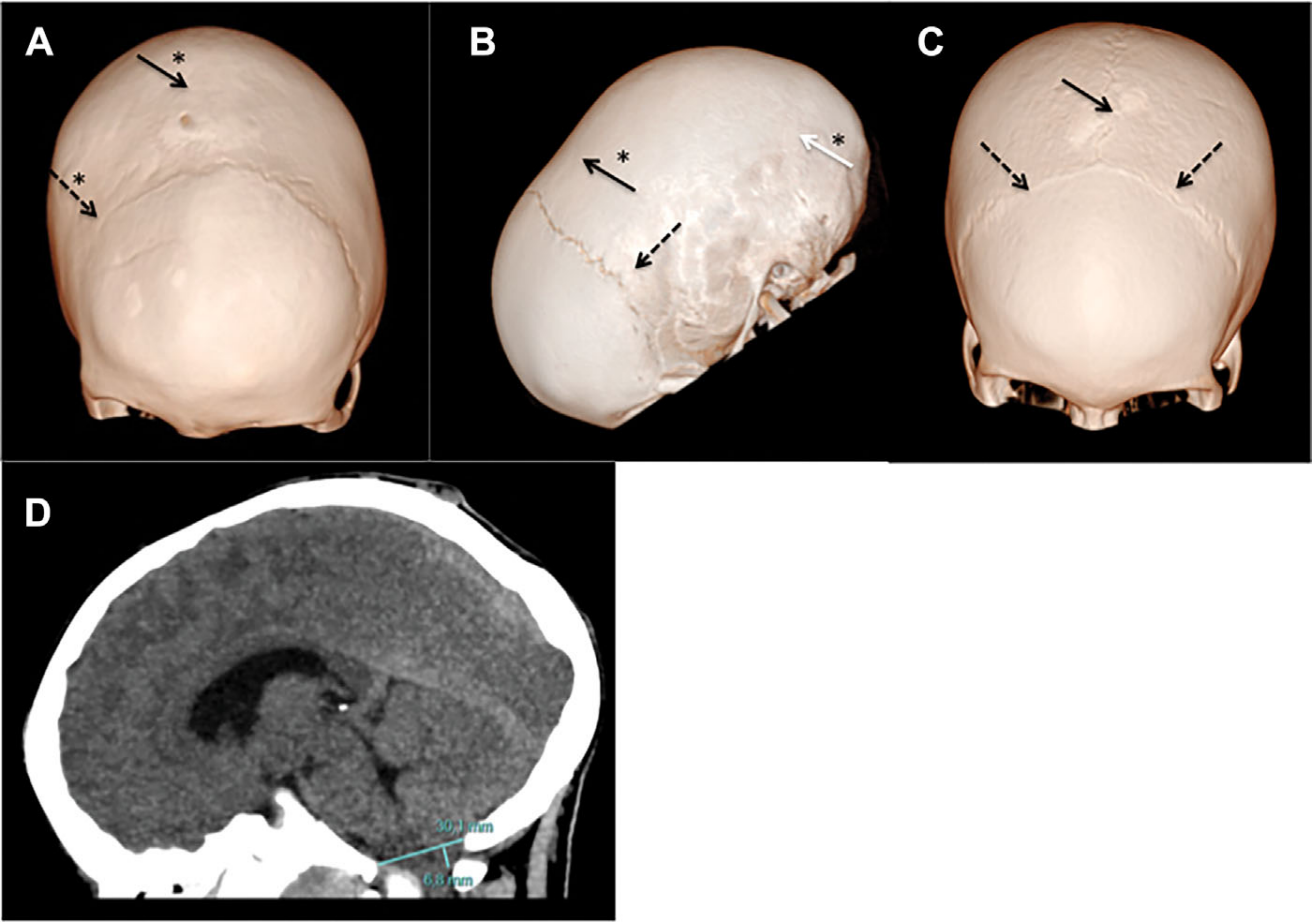
(*A*) An 8‐year‐old XLHR patient. Three‐dimensional CT skull bone window reconstruction. Complete closure of the sagittal and the right coronal sutures. The left coronal suture is patent but narrow. The patient also shows a congenital parietal foramina. (*B*) A 6‐year‐old XLHR patient. Three‐dimensional CT skull bone window reconstruction. Complete closure of the sagittal suture and both lambdoid sutures. (*C*) A 12‐year‐old XLHR patient. Three‐dimensional CT skull bone window reconstruction. Patent sagittal and coronal sutures. All very narrow. (*D*) An 8‐year‐old XLHR patient. Head CT sagittal slice parenchyma window showing a 6.8‐mm descent of the cerebellar tonsils. Continuous black arrows indicate sagittal suture. Discontinuous black arrows indicate coronal sutures. Continuous white arrow indicates lambdoid suture. Asterisks indicate closed sutures.

In 2 children with a complete fusion of the sagittal suture, the right coronal suture was also partially affected with a plagiocephalic deviation of the forehead and orbital bandeau (Fig. 2 *A*).

In another child, a closure of both lambdoids was associated with the loss of the sagittal suture (Fig. 2 *B*). The remaining 41 patients displayed patent lambdoid and coronal sutures.

However, even when sutures were found to be patent, these were found to be abnormally narrow.

Patients with craniosynostosis had a smaller CI in comparison to patients without craniosynostosis, 74 ± 5.4 (range 65 to 84) versus 78 ± 4.2 (range 72 to 90) (*p* = 0.03).

Children with sagittal suture loss either partial or complete had a greater frontal cranial vault bone thickness in comparison to those with a patent suture (9.64 mm versus 7.44 mm, *p* = 0.002).

The visibility (versus compression) of the sulci was reduced in children with the craniosynostosis (*p* = 0.044).

The sella turcica was significantly larger in its anteroposterior diameter in children with total or partial closure of the sagittal suture in comparison to those without (8.12 mm versus 7.09 mm, *p* = 0.04). The height of the sella turcica on sagittal reconstructions was similar in patients with patent and closed sagittal sutures.

None of the children displayed a ventricular dilation.

Craniosynostosis was significantly associated with a history of dental abscesses (*p* = 0.035, chi‐square test). However, we found no correlation with ALP or leg bowing at the time of CT scan evaluation (Table 3).

**Table 3:** Craniosynostosis and Chiari I Malformation–Associated Clinical and Biochemical Disease Characteristics

	*n*	Abscesses	CI <80	Chiari I	ALP >400 (IU/L)	Leg bowing
No craniosynostosis	18	4 (22%)	14 (78%)	1 (5.5%)	8 (44%)	10 (55%)
Craniosynostosis	26	16 (61%)	22 (85%)	9 (35%)	7 (27%)	19 (73%)
Craniosynostosis						
No Chiari I	29	14 (48%)	24 (83%)	15 (52%)	18 (62%)	20 (69%)
Chiari I	10	6 (60%)	9 (90%)	9 (90%)	9 (90%)	9 (90%)

CI = cranial index; ALP = alkaline phosphatase.

### Cranial index

We found a cranial index <75% and mesocephaly in 16 (36%) and 18 (41%) patients, respectively. The remaining 10 (23%) patients had a normal cranial index >80%.

Of the 16 children with a skull deformity, 13 and 1 had a complete or partial fusion of the sagittal suture, respectively.

Interestingly, of 10 patients with a cranial index >80%, 6 had craniosynostosis, with complete fusion of the sagittal suture in 5 patients and partial fusion in 1 patient.

Overall, 8 patients with a complete fusion of the sagittal suture did not have a cranial index under 75%.

### Chiari type 1 malformation (Table 4)

The position of the cerebellar tonsils was assessed in all but 5 patients in whom the radiological interpretation was not possible.

On sagittal and coronal reconstructions, a clear descent of cerebellar tonsils was found in 25% of the children (7 >5 mm and 3 <5 mm) (Table 4 and Fig. 2 *C*). The bulbo‐medullary junction was under the foramen magnum in 9 of these 10 XLHR children. The bulbo‐medullary junction was always above the foramen magnum in XLHR children without any cerebellar tonsil descent.

**Table 4:** Clinical, Biochemical, and Radiological Characteristics of Patients With Chiari I Malformations

Chiari I malformation and protrusion of the cerebellar tonsils	>5 mm *n* = 7	<5 mm *n* = 3	No protrusion *n* = 29	N/A *n* = 5
Sagittal suture (*n*)				
Complete fusion	5	2	9	2
Partial fusion	1	1	5	1
Patent	1	0	15	2
Cranial index				
<75%	4	0	9	3
75–80%	2	3	5	0
>80%	1	0	15	2
Leg deformities (*n*)				
Yes	6	3	18	2
No	1	0	11	3
Dental abscesses (*n*)				
Yes	4	1	13	3
No	3	2	16	2
ALP (*n*)				
<400 IU/L	4	1	19	3
>400 IU/L	3	1	10	1
N/A	0	1	0	1

ALP = alkaline phosphatase.

Nine of 10 children displayed a fusion of the sagittal suture in addition to the Chiari I malformation. The closure was complete in 7 and partial in 2 children. Intriguingly, one child aged 2.7 years with an overt patent sagittal suture had a Chiari I malformation. Closure of the sagittal suture was associated with the descent of the cerebellar tonsils (*p* = 0.03, chi‐square test).

The Chiari I malformation was diagnosed at a mean age of 7.0 ± 3.0 years (*n* =10, range 2.7 to 12.6 years). De novo and familial cases were equally affected. The diagnosis of Chiari I malformation did not correlate with the history of dental abscesses, ALP, or leg bowing (Table 3). Four of 10 patients with a Chiari malformation required neurosurgery.

## Discussion

This retrospective study of 44 children affected with XLHR highlights the high incidence of cranial vault and craniovertebral anomalies in this rare disease.

Our study shows that sagittal suture fusion is frequent in children with XLHR, ie, 60%, and that in some patients other sutures may also be affected, such as the coronal or the lambdoid sutures. However, in these latter patients, the sagittal suture was also concomitantly fused. We found a greater incidence of sagittal suture fusion than that reported in previous studies. Reilly and colleagues found 16 cases of craniosynostosis out of 59 rachitic children examined (27%).^16^ More recently, Currarino and colleagues found sagittal synostosis in 13 of 28 patients, all younger than 18 years, with hypophosphatemic rickets (46%).^25^ Our findings may be explained by the methodology used, which was based on head CT scans, allowing for a detailed analysis of the suture patency.

To have robust results in this study, we chose to use strict criteria to define partial and complete suture closure. However, the subjective opinion of the radiologists was that even patent sutures were abnormally narrow. Unfortunately, no reference data on normal coronal and sagittal suture widths exist for comparison.

In our series of 44 XLHR children, those with synostosis also presented an altered compliance of the skull with reduced visibility of the sulcei more commonly than those with open sagittal sutures (Fig. 2 *D*). Despite the fusion of the sagittal suture, the cranial indexes were only minimally affected. Although a systematic craniofacial clinical quantitative assessment was not performed, the physicians involved in this study (AR, JB, AL, and FdR) reported at most mild dolichocephaly in these XLHR children. This finding probably explains why the fusion of the sagittal suture has been underdiagnosed in the past. The closure of the suture is probably a late event in the growth of the skull of children with XLHR and occurs at a time when the overall shape of the skull, within normal limits, has already been achieved. Nevertheless, the cranial index tends to be lower in children with sagittal fusion compared with those without.

Interestingly, children with dolichocephalic skulls with a visible sagittal suture on CT scans as well as children with absent sagittal suture but with a high cranial index were also identified in the study population. This finding highlights the limits of the clinical morphological examination of the skull in the prediction of suture status.

Similarly, a descent of the cerebellar tonsils, ie, Chiari I malformation, was commonly found in our XLHR patients. About 20% of the investigated children met the criteria for Chiari I malformation. This finding is in accordance with a previous MR‐based study of 16 patients with hereditary hypophosphatemic rickets by Caldemeyer and colleagues, who found a Chiari I malformation in 7 of 16 patients.^26^ In the Caldemeyer study, 1 patient presented with clinical neurological symptoms, and 1 patient complained of nonspecific subjective signs. This limited or absent symptomatology was also found in our group of patients. Even though 4 patients with a Chiari malformation required neurosurgery (40%), only 2 patients in our study were symptomatic (described in Results). The Chiari I malformation was significantly associated with a fusion of the sagittal suture in most patients, suggesting that the cerebellar descent could be due to the reduced compliance of the overall skull during encephalic growth. This hypothesis is reinforced by the reduced size of subarachnoid spaces found in children with XLHR and Chiari I malformation. The cranial index being smaller in children with XLHR and Chiari I malformation confirms that the timing of suture closure also plays a role in the compliance of the posterior fossa.

Leg bowing and elevated ALP levels were more frequent in XLHR children with Chiari I malformation. Leg bowing was also found to be more common in children with craniosynostosis. The association between clinical and biological criteria of severity (which reached statistical significance only for history of dental abscesses) and radiological findings suggests that craniosynostosis and Chiari I malformation are features of severe forms of XLHR. This finding can only stress the importance of early diagnosis and adequate treatment of children with XLHR patients in order to try to avoid the development of these conditions.

In our series of XLHR patients, the association of Chiari I malformation and sagittal suture fusion was extremely common. This contrasts to the low incidence of Chiari I malformation in patients with isolated scaphocephaly.^27^ This discrepancy identified in XLHR patients suggests that the natural history of synostosis and descent of the cerebellar tonsils is likely different in XLHR compared with patients with isolated scaphocephaly or isolated Chiari I malformation. It also challenges the extrapolation of natural history, prognosis, and surgical indications from one condition, eg, isolated Chiari I malformation, to another, ie, XLHR.

A study in adults with XLHR is now mandatory to assess the prevalence and the long‐term outcomes of cranial vault and craniovertebral anomalies.

Our study highlights the possible role of phosphate and calcium metabolism dysregulation in the development of synostosis or Chiari I malformation. These metabolic disorders may be underestimated as causing factors. Screening for hypophosphatemia and elevated ALP should be recommended in children with late‐onset sagittal synostosis and in children with Chiari I malformation independently from the overall shape of their skull and cranial index. Obviously, this screening should be mandatory whenever the Chiari I malformation is associated with a dolichocephalic skull. Further studies are needed to estimate whether genetic screening of *PHEX* and *FGF23* could be useful in patients with Chiari I malformations to improve our understanding of this condition.

Our study has several limitations. We collected data retrospectively, which explains the lack of quantitative clinical assessment such as cranial circumference and thorough neurological investigations to identify putative consequences of the Chiari I malformation and altered skull compliance. CT scans were performed during the routine follow‐up of patients; therefore, this study cannot evaluate the age of onset of synostosis and cerebral descent nor their evolution throughout time.

Taking into account radiation exposure using CT scans in children, screening for both Chiari I malformations and craniosynostosis could be performed using MR imaging. However, the best MR sequences for suture analysis, which seem to be the so‐called “black bone sequences,” are still under study. The gold standard for the analysis of bones and sutures still remains the CT scan with 3D reconstructions, which at present delivers minimal X‐ray exposure.

This study highlights that sagittal suture fusion, ie, craniosynostosis and Chiari I malformation, are two common complications that are associated with the severity of disease in children with XLHR. Sixty‐one percent of the children showed either craniosynostosis and/or a Chiari I malformation. Even though only 2 patients had neurological symptoms, 4 required neurosurgery (15% of patients with abnormal imaging). This study demonstrated that a radiological evaluation should be considered in XLHR children if a proper diagnosis is warranted because cranial vault and craniovertebral anomalies are underestimated by clinical evaluation only.

We thus suggest systematic thorough neurological examination during follow‐up of children with XLHR and think that neuroimaging should be considered.

Further studies are needed to better assess the short‐term and long‐term clinical impact of these complications in this population. Nevertheless, the association of neurosurgical features confirms the importance of a regular systematic multidisciplinary evaluation and care of these patients.

## Disclosures

All authors state that they have no conflicts of interest.
